# Potential of IL-33 for Preventing the Kidney Injury via Regulating the Lipid Metabolism in Gout Patients

**DOI:** 10.1155/2016/1028401

**Published:** 2016-08-07

**Authors:** Lihua Duan, Yan Huang, Qun Su, Qingyan Lin, Wen Liu, Jiao Luo, Bing Yu, Yan He, Hongyan Qian, Yuan Liu, Jie Chen, Guixiu Shi

**Affiliations:** ^1^Department of Rheumatology and Clinical Immunology, The First Hospital of Xiamen University, Xiamen 361003, China; ^2^Department of Immunology, Basic Medicine Science, Medical College, Xiamen University, Xiamen 361005, China

## Abstract

Interleukin-33 (IL-33), the most recently discovered member of the IL-1 superfamily, has been linked to several human pathologies including autoimmune diseases, sepsis, and allergy through its specific IL-1 receptor ST2. However, there is little information regarding the role of IL-33 in gout. In this study, we investigated the potential role of IL-33 in gout patients. The serum level of IL-33 was measured by ELISA, and the clinical and laboratory parameters, serum creatinine, urea, and lipid, were extracted from medical record system. The serum IL-33 expression was predominantly increased in gout patients compared to healthy controls, and the IL-33 levels were higher in patients without kidney injury. Furthermore, IL-33 showed a negative correlation with biomarkers of kidney injury, such as CRE and urea. The lipid metabolism dysfunction, tophi, and hypertension are the common reasons for kidney injury in gout. Interestingly, inverse and positive correlation of IL-33 expression was observed in LDL and HDL, respectively. However, there was no significant alteration in the gout patients with hypertension and tophi. These data suggested that IL-33 might act as a protective role in kidney injury through regulating the lipid metabolism in gout.

## 1. Introduction

Gout is the most common metabolic disease in human, which results from the purine metabolism disorder [1]. Due to loss of uricase in human, purine metabolism in human and apes is completely different form that in other mammalian species. Monosodium urate (MSU) crystals formation results from serum uric acid concentrations rise above the physiological saturation, which deposit in joints and soft tissues leading to arthritis, soft tissue masses, nephrolithiasis, and urate nephropathy [2]. It is reported that 75%–80% of gout is associated with metabolism disorder. More than 80% of the patients with hyperlipoproteinemia are accompanied with hyperuricaemia [3]. The finding of hyperlipidaemia in gout patients is common, including an increase in small dense LDL cholesterol and a reduction in HDL cholesterol [4]. It was shown that metabolic syndrome including high levels of LDL and TG might be an important factor in the cause of chronic kidney disease (CKD) [5, 6]. The pathogenesis of gout was often accompanied by lipid metabolic dysregulation. Previous study has shown that excess triglyceride-rich lipoproteins are oxidized by oxidative stress in chronic kidney disease. The prevention of hypertriglyceridemia is one of the most important steps for the treatment of chronic kidney disease [7]. Therefore, lipoprotein metabolic disorder might contribute to the kidney injury in gout patients.

IL-33 is a member of the IL-1 cytokine family, and the intracellular pathway of IL-33 signaling is similar to that of IL-1 signaling [8]. The IL-1 family of cytokines includes IL-1*α*, IL-1*β*, and IL-18 [9]. IL-33 signaling occurs by binding to ST2, IL-1RAcP, subsequent intracellular signaling pathways including MyD88, IRAK (IRAK1, IRAK4), and TRAF6, leading to the activation of NF-*κ*B and MAPKs (p38, JNK, and ERK) pathways [10, 11]. IL-33 production is upregulated in inflamed tissues and acts as an early inducer of inflammation contributing to the further amplification of inflammatory responses [12]. Furthermore, recent studies have demonstrated that IL-33 played a critical role in obesity-associated inflammation, atherosclerosis, and metabolic abnormalities through promoting the production of T helper type 2 (Th2) cytokines and polarizing macrophages towards a protective alternatively activated phenotype.

Although the detrimental role for IL-33 in the RA and SpA has been reported, the role of IL-33 in gout arthritis is still unknown. In the present study, we examine serum levels of IL-33 by ELISA from gout patients, and correlation with clinical markers in patients with gout was analyzed. We found that serum levels of IL-33 in gout patients were significantly higher than healthy subjects. Particularly, the IL-33 levels were higher in patients without kidney injury. Previous studies showed that IL-33 exacerbates acute kidney injury, whereas IL-33 showed a negative correlation with biomarker of kidney injury, such as CRE and urea in gout patients. There were no significant differences in the gout patients with tophi or hypertension which can cause kidney damage. However, an inverse correlation of IL-33 expression was observed in LDL, and a significantly positive correlation was observed in HDL. These data suggested that IL-33 might prevent the kidney injury through regulating the lipid metabolism in gout patients.

## 2. Materials and Methods

### 2.1. Patients and Controls

A total of 41 patients diagnosed with gout (2 women and 39 men, age 26–91, mean 58.3 years) were recruited from the outpatient clinic and ward of the Department of Rheumatology and Clinical Immunology, the First Hospital of Xiamen University. All patients were diagnosed based on the American College of Rheumatology classification criteria [13]. The results were compared with a population of 44 healthy volunteers (healthy controls) matched for sex and age. Local ethics committee approved the study and informed consent was obtained from patients and control subjects. Clinical data and several laboratory parameters, such as CRE, urea, HDL, LDL, and UA, of patients were gathered. The number and clinical characteristics of healthy controls and patients with gout were summarized in Table 1.

### 2.2. Detection of Cytokines by ELISA

Four milliliters of blood was collected in sterile coagulant tubes and was then centrifuged at 3,500 rpm for 5 min at ambient temperature to obtain serum, which was immediately frozen and stored at −80°C until batch analysis. Concentrations of IL-33 (PeproTech, USA) were determined by ELISA kits according to the manufacturers' protocols.

### 2.3. Statistical Analysis

All data were analyzed in GraphPad Prism5. Results are presented as mean ± SEM. The Mann-Whitney *U* test and Spearman's correlation analysis were used to calculate significance. Statistical significance was accepted for *p* values < 0.05.

## 3. Results

### 3.1. Clinical Characteristics of Gout Patients

The clinical characteristics of gout patients (see Table 1) were summarized for this study. Forty-one patients with gout and forty-four healthy controls of Southern Chinese population were enrolled. The mean age for gout patients was 58.3 years with range (26–91); there were 39 males and 2 females. The mean course of the disease was for 10.6 years with range of 0.3–40 years. Among these 41 patients, 36 patients (87%) had acute phase, 15 patients (36.5%) had tophi in Gout, 27 patients (65.8%) had hyperuricemia, 18 patients (43.9%) had hypercholesteremia, 21 patients (51.2%) had hypertension, 13 patients (31.7%) had gouty kidney damage, 19 patients (46.3%) had osteoporosis, and 4 (9.7%) had fever.

### 3.2. Serum IL-33 Levels Are Increased in Gout Patients

**Figure 1:**
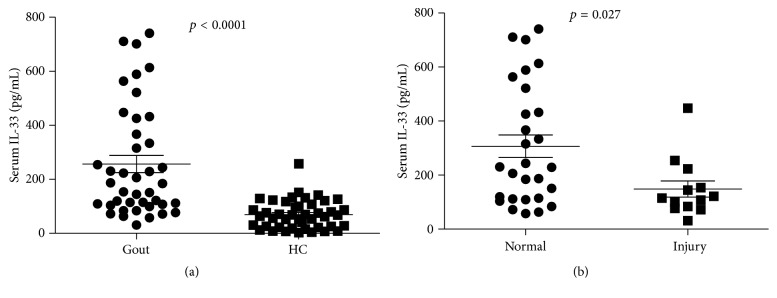
Increased IL-33 expression in gout patients with renal damage. (a) The serum levels of IL-33 in gout patients (*n* = 41) and healthy controls (HC) (*n* = 44) were detected by ELISA; each symbol represented an individual sample and horizontal lines showed median values. (b) The gout patients were divided into two groups, one group with kidney function injury (*n* = 13) and one without (*n* = 28). Mann-Whitney *U* test was conducted to compare the data between two groups.

It has been demonstrated that IL-33 played a critical role in many diseases. However, the role of IL-33 in gout patients has not been described until now. Currently, compared with healthy controls, the serum IL-33 levels in gout patients were significantly increased (*p* < 0.0001). As shown in Figure 1(a), the median level of IL-33 in gout patients was 184.6 pg/mL with range of 31.3–741.3 pg/mL, while healthy control was 65.6 pg/mL (2.16–257.8 pg/mL). Furthermore, we observed that the gout patients with renal injury showed lower IL-33 levels (median 114.9 pg/mL: 31.3–447.8) when compared with gout patients without renal injury (median 299.5 pg/mL: 57.9–741.3) (*p* = 0.027) (Figure 1(b)).

### 3.3. Association of IL-33 Expression in Gout Patients with Tophi, Hypertension, and HDL

**Figure 2:**
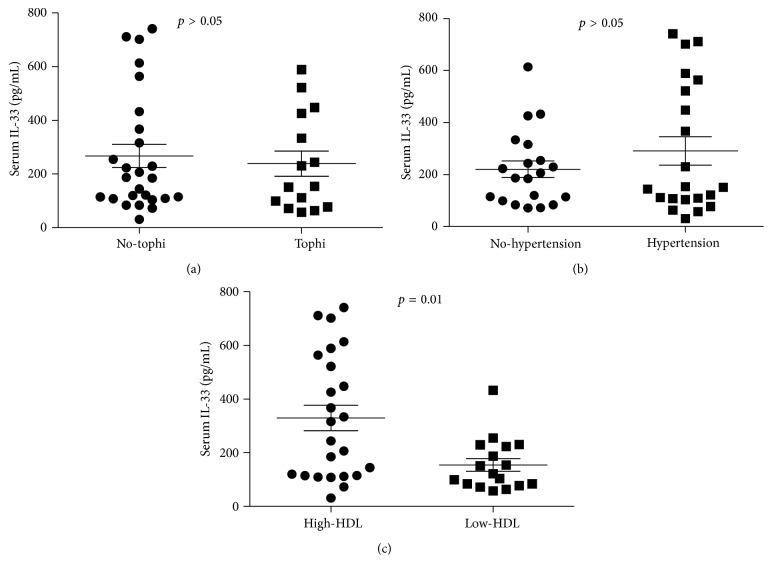
Difference of IL-33 expression in gout patients with tophi, hypertension, and HDL. The renal function was affected by tophi, hyperlipoidemia, and hypertension in gout patients. The serum levels of IL-33 in patients were grouped according to tophi, hypertension, and HDL. These groups were evaluated by Mann-Whitney *U* test.

The renal function in gout patients was often damaged by tophi, hyperlipoidemia, and hypertension. Gout patients were grouped according to tophi, hypertension, and HDL. The IL-33 expression of gout patients with tophi (median 154.0 pg/mL: 57.9–589.1) or not (median 185.7 pg/mL: 31.3–741.3) (*p* = 0.635) and with hypertension (median 150.7 pg/mL: 31.3–741.3) or not (median 196.7 pg/mL: 71.7–614.0) (*p* = 0.804) did not show a significant difference (Figures 2(a) and 2(b)). Interestingly, when compared with low serum HDL levels, the gout patients with high-HDL levels showed an increased IL-33 expression (328.9 ± 231.5 versus 154.2 ± 97.35 pg/mL, *p* = 0.01).

### 3.4. Correlation of Serum IL-33 Levels with Kidney Function Indictors in Gout Patients

**Figure 3:**
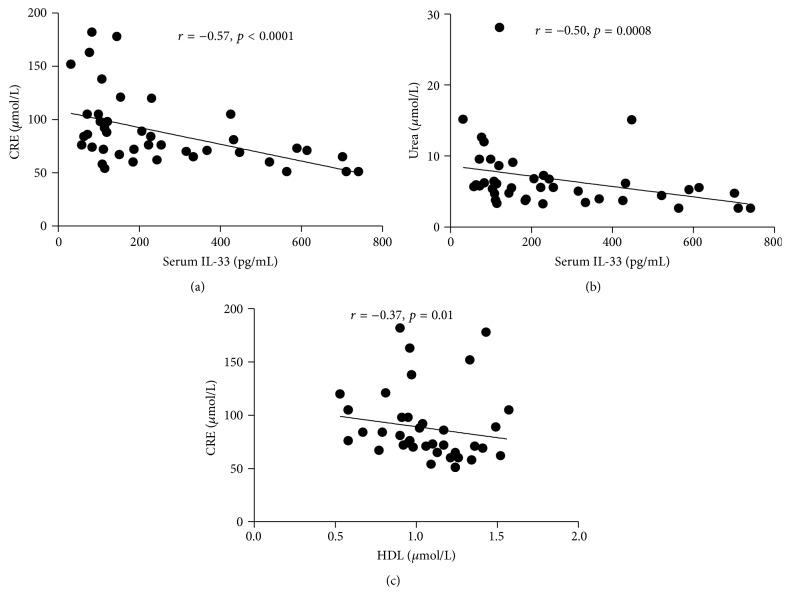
Negative correlation of IL-33 with CRE and urea in gout patients. (a, b) The determination of linear relationships between IL-33 expression and CRE and urea in gout patients was performed by Spearman correlation coefficient. (c) Serum HDL levels showed a negative correlation with Cre. Spearman's correlation analysis was used to calculate significance.

In addition, the role of IL-33 in the gout patients with kidney injury was analyzed. Spearman's correlation coefficient was used for the assessment of correlation between IL-33 levels and CRE or urea. As expected, the IL-33 levels were negatively correlated with CRE (*r* = −0.57, *p* < 0.0001) and urea (*r* = −0.49, *p* = 0.0008) (Figures 3(a) and 3(b)). The above results showed that HDL played a protect role in the development of the kidney injury in gout patients. Here, a negative correlation between HDL and CRE was depicted in Figure 3(c).

### 3.5. Potential of IL-33 in the Modulation of Lipid Metabolism

**Figure 4:**
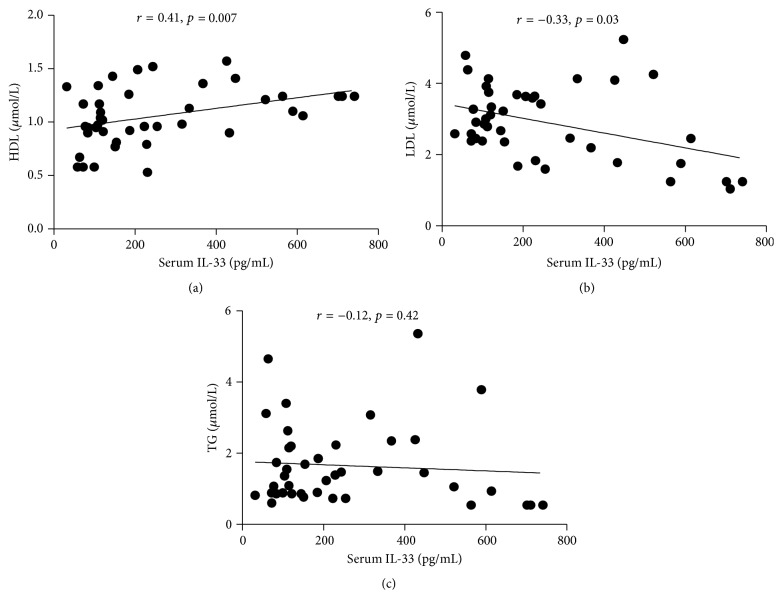
The correlation of IL-33 with lipoprotein in gout patients. (a) Serum IL-33 was positively correlated with HDL in gout patients (*r* = 0.41, *p* = 0.007). (b, c) Serum IL-33 level was negatively correlated with LDL (*r* = −0.33, *p* = 0.03) and TG (*r* = −0.12, *p* = 0.42) in gout patients. Serum IL-33 level was not detected to correlate with TG from gout patients. Spearman correlation analysis was conducted.

Furthermore, lipid metabolism disorder often occurred in gout patients, which caused kidney damage (Figure 3(c)) in gout patients, and recent study has shown that IL-33 plays a protective role in atherosclerosis. Thus, the correlation of IL-33 with lipid was analyzed. We found that a positive correlation between IL-33 and HDL (*r* = 0.41, *p* = 0.007) was observed (Figure 4(a)). Consistently, LDL, a critical lipid for the development of atherosclerosis, was in negative correlation with IL-33 (*r* = −0.33, *p* = 0.03) (Figure 4(b)). A negative correlation between IL-33 and TG was observed, although there is no significant difference (*r* = −0.12, *p* = 0.42) (Figure 4(c)).

## 4. Discussion

In the present study, we demonstrated the IL-33 expression and its potential role in gout patients. Serum IL-33 levels were significantly elevated in patients with gout patients when compared with healthy control subjects. Furthermore, the elevated IL-33 levels were considerably reduced in renal impairment when compared with normal renal function in gout patients. There were no significant differences whether the gout patients had tophi or hypertension which can cause kidney damage. However, an inverse correlation of IL-33 expression was observed in LDL, and a significantly positive correlation was observed in HDL. These data suggested that IL-33 might prevent the kidney injury through regulating the lipid metabolism in gout patients.

It was first demonstrated that IL-33 activates T helper type 2 (Th2) cells and mast cells to secrete Th2 cell-associated proinflammatory cytokines and chemokines [8]. Additionally, IL-33 can also induce proinflammatory effects depending on Th1/Th17 immune response [14, 15]. Previous studies have reported that IL-33 acts as an endogenous “danger signal” or “alarmin” that may alert the immune system in response to inflammatory diseases [16], such as hypersensitive diseases like asthma [17], autoimmune diseases like rheumatoid arthritis [18], allergic rhinitis [19], and autoimmune conjunctivitis [20]. IL-33 from necrotic cells might induce autophagy, which can further balance the effects of increased apoptosis secondary to contrast-induced nephropathy in diabetic kidney disease [21]. In addition, IL-33 promotes acute kidney injury through CD4 T cell-mediated production of CXCL1 [22]. Although the cytokine secretion by macrophage can be enhanced by IL-33 [23–25], in our study IL-33 was found increased in gout patients with no kidney injury. The reason for the dual function of IL-33 might be the various inflammatory environments in different diseases.

It is well known that gout is associated with cardiovascular and metabolic diseases, such as hypertension, hyperlipidaemia, and diabetes mellitus. 75%–80% of gout patients had combined high blood lipoprotein [4, 26]. Interestingly, it has been demonstrated that IL-33 is a potent inhibitor of macrophage foam cell formation in vivo and in vitro and attenuates atherosclerosis. IL-33 blocks foam cell formation by directly regulating the expression of genes for AcLDL/OxLDL uptake and storage of cholesteryl esters and triglycerides and cholesterol efflux/transport and by inducing a phenotypical Th1-to-Th2 switch [27]. IL-33 may play a protective role in the development of atherosclerosis via the induction of IL-5 and ox-LDL antibodies [28]. Low serum high-density lipoprotein cholesterol level was an independent predictor for gouty flares [29], while gout patients who had higher high density lipoprotein (HDL) levels with metabolic syndrome were at the lowest risk [30]. In line with above results, we also observed that serum levels of IL-33 were positively correlated with HDL from gout patients, and the expressya ion of IL-33 was markedly high in the patient without kidney damage. As expected, serum IL-33 levels showed an inverse correlation with CRE, urea. and UA from gout patients.

MSU has been identified as an endogenous factor for kidney damage [11]. NLRP3 activation induced by MSU crystal triggers caspase-1-dependent IL-1*β* and IL-18 secretion, which induces a general inflammatory response including recruitment of neutrophils and macrophages to the site of crystal formation. These cytokines and infiltrated immune cells in the kidney interstitial tissue result in the renal tubular epithelial cell damage and interstitial fibrosis, and the inflammation condition is easy to cause the glomerulitis and renal dysfunction. Although the cytokine secretion by macrophage can be enhanced by IL-33 [23–25], in our study gout patients with no kidney injury showed a high IL-33 expression. A number of epidemiological studies have reported that serum uric acid levels are the high-risk factor in the pathogenesis of hypertension, and high plasma uric acid levels are common in patients with arterial hypertension. Hypertension is commonly associated with renal vasoconstriction, which also leads to kidney injury, but no correlation between hypertension and IL-33 expression was observed in this study. Furthermore, the spectrum of renal diseases in gout patients includes urate stones, acute uric acid nephropathy, and chronic urate nephropathy. Chronic urate nephropathy may be associated with a series of complicating factors, rather than simply with excessive depositions of uric acid within renal tissues, such as microvascular damage from untreated hypertension. There was also no difference caused by the depositions of uric acid. Thus, IL-33 might play a protective role in the kidney injury of gout through regulating lipid metabolism.

In conclusion, the results presented here suggest that the serum IL-33 could be a sensitive marker for kidney function in gout patients. Interestingly, it is noted that IL-33 was associated with HDL and CRE in gout, suggesting that the IL-33 may play a beneficial role in the pathogenesis of gout. Of course, further studies are required to explore the specific regulation mechanism between IL-33 and renal function. These data maybe further suggest a novel approach in treating gouty arthritis.

## Figures and Tables

**Table 1:** Demographic data and clinical characteristics of subjects in the study.

Characteristics	Gout patients	Healthy control
Total	41	44
Male sex	39 (95%)	41 (93%)
Age at study mean (SD) years	58.3 (15.55)	56.8 (17.91)
Course of disease, mean (range) years	10.6 (0.3–40)	—
Clinical manifestation, *n* (%)		
Hyperuricemia	27 (65.8%)	—
Acute phase	36 (87%)	—
Tophi	15 (36.5%)	—
Gouty kidney damage	13 (31.7%)	—
Hypercholesteremia	18 (43.9%)	—
Hypertension	21 (51.2%)	—
Osteoporosis	19 (46.3%)	—
Fever	4 (9.7%)	—
